# Case Report: Two cases of chemotherapy refractory aggressive variant prostate cancer with extreme durable response to PARP inhibitor

**DOI:** 10.3389/fonc.2025.1533627

**Published:** 2025-04-24

**Authors:** Bohao Jiang, Yifan Bi, Yiming Chen, Jianbin Bi, Jian Deng, Gejun Zhang

**Affiliations:** ^1^ Department of Urology, The First Hospital of China Medical University, Shenyang, Liaoning, China; ^2^ Third Department of Medical Oncology, The Fifth People Hospital of Shenyang, Shenyang, Liaoning, China

**Keywords:** prostate cancer, aggressive variant prostate cancer, treatment, PARP inhibitor, chemotherapy, radiotherapy

## Abstract

**Background:**

Aggressive variant prostate cancer (AVPC) represents a distinct clinical subset characterized by resistance to novel hormone therapies and an unfavorable prognosis, frequently associated with the concurrent loss of tumor suppressor genes (TSG) such as PTEN, RB1, and TP53. While the progression-free survival (PFS) and overall survival (OS) of AVPC are relatively short, the optimal first-line treatment remains unclear.

**Presentation:**

In this case report, we presented two *de novo* AVPC cases who have ultimately benefited from the usage of PARP inhibitors. The first patient was a 64-year-old male who was diagnosed during prostate biopsy featured by mutations in PTEN, and loss of RB1, BRCA2, ATM, and FANCA. He was treated with docetaxel/albumin-bound paclitaxel and cisplatin in the first line. Second-line therapy was applied with radiotherapy and Olaparib after failure of first-line therapy, resulting in a PSA response sustained for three years. The second case was a 75-year-old male with localized neuroendocrine feature and mutations in TP53, loss of RB1 and HDAC2. He was treated with sustained ADT and chemotherapy in the first-line treatment. Radiotherapy and Fluzoparib + abiraterone was applied as subsequent treatments with a PSA response for 2 years.

**Conclusions:**

These two cases demonstrating a satisfactorily durable response to PARP inhibitors indicating its clinical benefit in AVPC population with detected DNA damage response (DDR) defects. The survival improvement with PARP inhibitors observed in our clinical experiences, along with current advances in tumor sequencing provide more information on future clinical trials and explorations of innovative therapies in AVPC population.

## 1 Introduction

Prostate cancer, counting for 1466680 new cases (14.2%) and causing 397430 deaths (9.7%) in 2022, ranks as the second most common malignant tumor in males according to the data reported by the International Agency for Research on Cancer (IARC) (1). Androgen deprivation therapy (ADT) remains the standard treatment for high-risk localized or advanced prostate cancer. However, this therapy may fail in certain subtypes, such as aggressive-variant prostate cancer (AVPC) (2–6). It is often featured with gene mutations such as TP53 and RB1 deficiency and a loss of androgen dependence resulting in a poor prognosis of progression-free survival (PFS) and overall survival (OS) (7, 8). While hormone-related therapy may have a poor therapeutic effect, the optimal treatment for AVPC remains unclear, highlighting an urgent need for exploration of effective therapies.

Recent next-generation sequencing (NGS) studies have revealed the genomic landscape of prostate cancer and identifying commonly altered biological pathways, some of which may play crucial roles in determining the onset, progression and prognosis of the disease. The loss-of-function (LOF) alterations and deletions of some tumor suppressor genes, such as TP53, RB1 and PTEN, are often detected in AVPC. And they also serve as key genomic features in the diagnosis of neuroendocrine prostate cancer (NEPC), a specific subtype of NEPC with neuroendocrine characteristics (9). In this situation, the results of tumor sequencing can provide information for designing more precise therapeutic strategies. For example, the status of homologous recombination repair (HRR) genes is highly related with the response to PARP inhibitors and platinum-based chemotherapy (10). Several studies have demonstrated that PARP inhibitors show remarkable therapeutic effects on radiologic and biochemical improvement for patients with HRR defects, such as Olaparib approved by FDA in May 2020 (11). And the genomic features of AVPC and NEPC, which is similar with small cell carcinoma, provided evidences for the application of chemotherapy as first-line treatment (12).

Here we report the treatment experience and remarkable response to PARP inhibitors in 2 AVPC cases with next-generation sequencing results who undergone rapid progression after chemotherapy and ADT. They subsequently achieved remission through treatment with Olaparib and Fluzoparib separately. Adverse effects of the whole treatment process were graded according to the Common Terminology Criteria for Adverse Events (CTCAE) 5.0 standard.

## 2 Case presentation A (patient 1)

The first patient was a 64-year-old male, admitted with prostate pain and a high PSA level of 125 ng/ml in March 2021. After examination of magnetic resonance imaging (MRI) + diffusion-weighted imaging (DWI), he was suspected as a prostate cancer patient with pelvic lymph nodes and bone metastases. In April, the patient underwent a prostate biopsy of 10 cores and was originally diagnosed with prostate cancer. Further PET-CT + emission computed tomography (ECT) confirmed the existence of malignant pelvic lymph nodes on the left and multiple bone metastases. The patient was graded as cT4N1M1b with a Gleason Score 5 + 4. Due to the presence of extensive metastases and high clinical staging, we decided to choose systemic therapy rather than radical surgery. To evaluate the expected therapeutic effects of various drugs for systemic treatment, he undergone a next-generation genetic testing in which report 6 alterations in clinically relevant genes were revealed, including PTEN p.S227Cfs.Ter22, BRCA2 copy number loss, ATM copy number loss, RB1 copy number loss, CTNNB1 p.G34E, and FANCA copy number loss (**Table 1**).

**Table 1:** Genetic sequencing results of 2 AVPC cases and reasons for treatment selections.

Patient ID	Molecular mutations	Reasons for treatment selections
Case 1	PTEN p.S227Cfs.Ter22	First-line: Mutations of PTEN, RB1 showed a possibly poor effect of endocrine therapy; FANCA mutation showed a possible therapeutic effect of platinum-based chemotherapy.
	BRCA2 copy number loss	
	ATM copy number loss	
	RB1 copy number loss	Second-line: Mutations of BRCA2 and ATM suggest a relatively satisfactory effect of PARP inhibitors; Radiotherapy was taken due to the multiple distal metastatic foci.
	CTNNB1 p.G34E	
	FANCA copy number loss	
Case 2	TP53 p.G245D	First-line: Mutation of TP53 and RB1 showed a possibly poor effect of endocrine therapy. Its neuroendocrine feature suggested potential benefits of chemotherapy.
	FOXA1 p.H247R	
	FOXA1 p.M253_N256delinsl	
	FOXA1 p.N252del	Second-line: Without HRR gene mutations (BRCA1/2, ATM), PARP inhibitors were not taken at once. Radiotherapy was taken due to the multiple distal metastatic foci.
	FOXA1 p.R265_F266insS	
	RB1 copy number loss	
	HDAC2 copy number loss	
	RLCOL4 p.G543A	Third-line: PARP inhibitor was applied due to the mutation of HDAC2, which was reported potentially beneficial after failure of chemotherapy and radiotherapy.
	NF2 p.K387fs*39	
	COKN1B p.N124fs*20	

**Figure 1:**
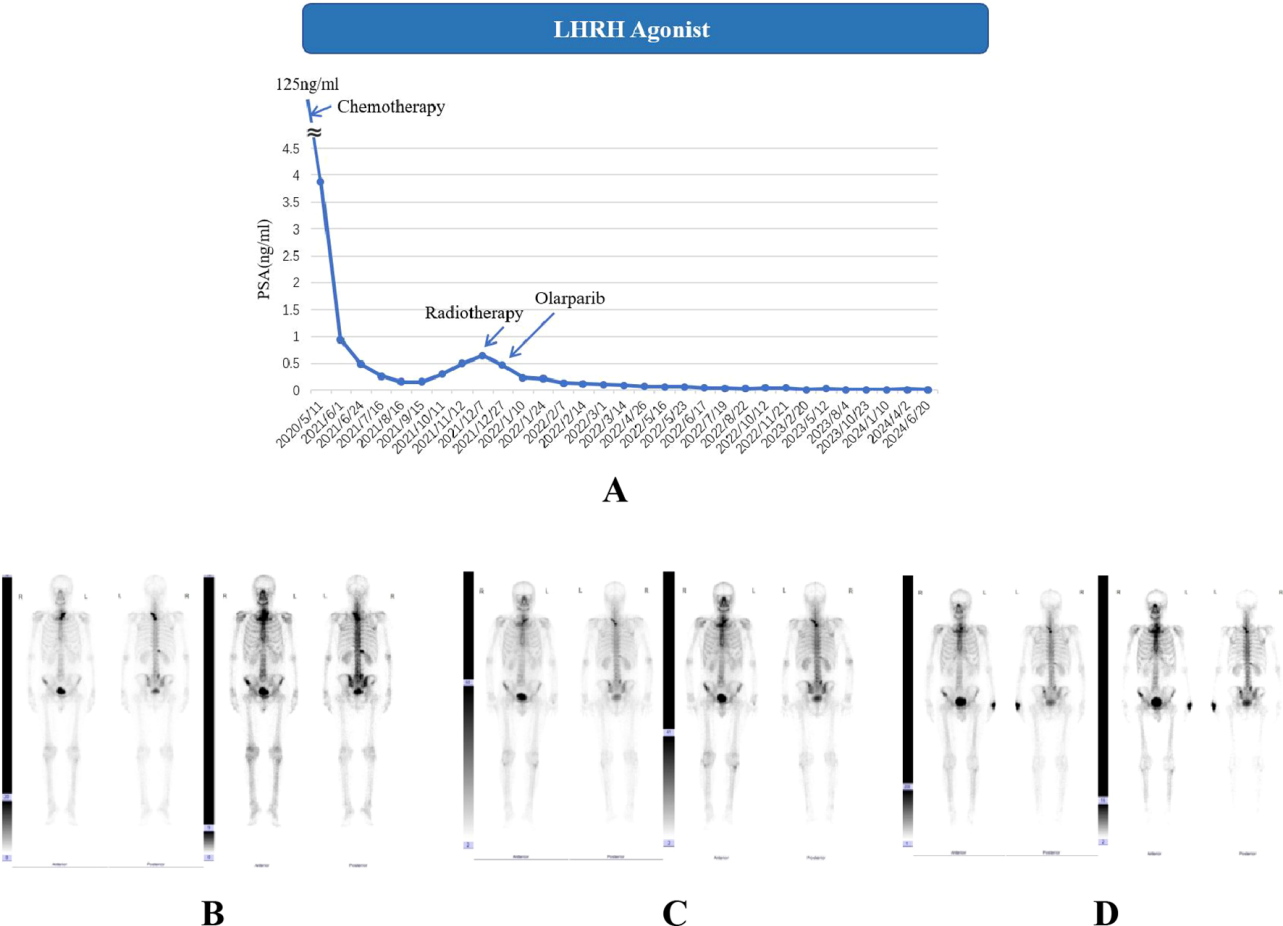
Serum PSA level **(A)** and ECT results **(B–D)** of case 1. **(A)**. LHRH: Luteinizing Hormone-Releasing Hormone. Chemotherapy: docetaxel/albumin-bound paclitaxel + cisplatin. The detailed dates and values of each PSA test can be found in the **Supplementary Table S2**; **(B)** ECT results for April 2021, radionuclide-concentrating foci were seen in T2-T3 vertebrae, right 12^th^ posterior rib, L5 vertebrae and bilateral iliac bones; **(C, D)**. The ECT results from November 2021 and November 2022 revealed no newly developed bone metastases.

As suggested in the genetic testing result, he was diagnosed as *de novo* AVPC and received continuous ADT (bicalutamide 50mg qd/goserelin 10.8mg) and chemotherapy as the first-line treatment. Regarding the specific chemotherapy drugs, he received docetaxel with cisplatin twice (docetaxel 120mg + cisplatin 120mg, intravenous drip). However, the patient expressed his concerns about the use of prednisone, which are often used in combination with docetaxel. As a result, the chemotherapy plan was changed into albumin-bound paclitaxel with cisplatin for four times (albumin-bound paclitaxel 400mg + cisplatin 120mg, intravenous drip). Detailed chemotherapy protocol was displayed in **Supplementary Table S1**. The serum PSA level dropped to 0.151 ng/ml after the 3-month chemotherapy (**Figure 1A**).

However, the PSA level doubled to 0.496 in December 2021, two months after the end of chemotherapy, indicating a biochemical progression of PCa (**Figure 1A**) without newly developed bone metastases in ECT images. As second-line therapy, one cycle of intensity modulated radiation therapy (IMRT) was applied with the planned target area set as prostate and seminal vehicle (75.6Gy/36f, 2.16Gy/f) and drainage area of obturator fossa, internal/external iliac and presacral lymph nodes (50.46 Gy/28f, 1.8Gy/f). Considering the circumstances of bone metastasis, an RT (35Gy/14f) was also performed on the skeletal metastatic area identified. After radiotherapy, the PSA level dropped to 0.237 ng/ml. At the same time as starting IMRT, the patient received continuous Olaparib use until now (300 mg, bid) due to the detection of BRCA2 alteration, successfully reducing PSA levels to 0.015 ng/ml and attaining a PSA response of 90% four months post-treatment, which is the most common PARP inhibitor in our clinical work with permission from China Food and Drug Administration (CFDA) (**Figure 1A**). The low PSA level remained stable throughout the subsequent treatment process. ECT monitoring showed no significant changes during follow-up (**Figures 1B–D**).

**Figure 2:**
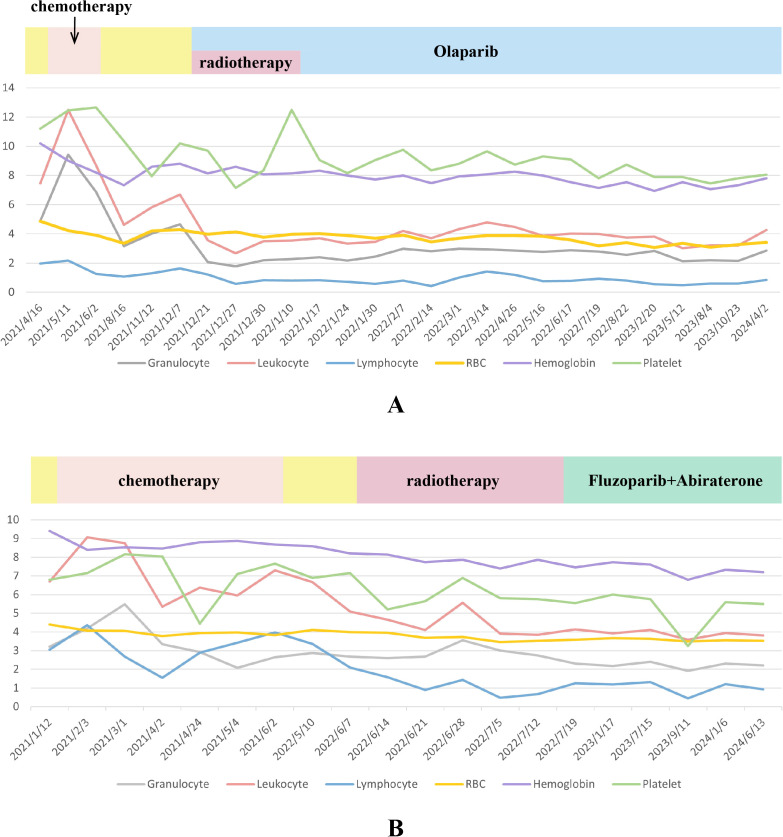
The trend of changes in various blood indicators during the entire treatment process of case 1 **(A)** and case 2 **(B)**. (1) Units for blood indicators: Granulocyte, Leukocyte, Lymphocyte, Platelet: 10^9^/L; Red blood cell (RBC): 10^12^/L; Hemoglobin: g/L. (2) In order to display the two indicators of hemoglobin and platelets on the same figure with the other four indicators, all hemoglobin values were divided by 15 and all platelet values were divided by 20.

For adverse events, the patient showed good tolerance throughout the entire treatment process during the monitoring of the patient’s blood cell counts (**Figure 2A**). Detailed numerical changes of each type of blood cell were presented in **Supplementary Figures S1**-**S6**. During the 6-month chemotherapy, only lymphocytopenia (grade 1) and anemia (grade 1) were observed. However, during IMRT + Olaparib period, occasional granulocytopenia (grade 1) and leukopenia (grade 1-2) were reported, together with relatively continuous lymphocytopenia (grade 1-2) and anemia (grade 1). There was only 1 time of grade 3 lymphocytopenia in the later period of IMRT.

## 3 Case presentation B (patient 2)

The second patient was a 75-year-old male with an elevated PSA level of 34.7 ng/ml. An ECT scan result revealed multiple foci of increased bone metabolism indicating a high possibility of malignant bone metastasis in L1 vertebrae, right iliac bone, pubic symphysis and left femur bone. Then, the patient underwent a prostate biopsy of 10 cores with a Gleason Score of 5 + 5 and expressions of synaptophysin in local leisions by immunohistochemical staining. He was originally diagnosed with metastatic hormone-sensitive prostate cancer in January 2021. The tumor was classified as T3bN1M1b, featuring local neuroendocrine prostate carcinoma (NEPC) with metastases of pelvic lymph nodes and bone. Genetic analysis of the biopsy tissues revealed 10 mutations in clinically relevant genes, including TP53 p.G245D, FOXA1 p.H247R, FOXA1 p.M253_N256delinsl, FOXA1 p.N252del, FOXA1 p.R265_F266insS, RB1 loss of copy number, HDAC2 loss of copy number, RLCOL4 p.G543A, NF2 p.K387fs*39, and COKN1B p.N124fs*20 (**Table 1**).

**Figure 3:**
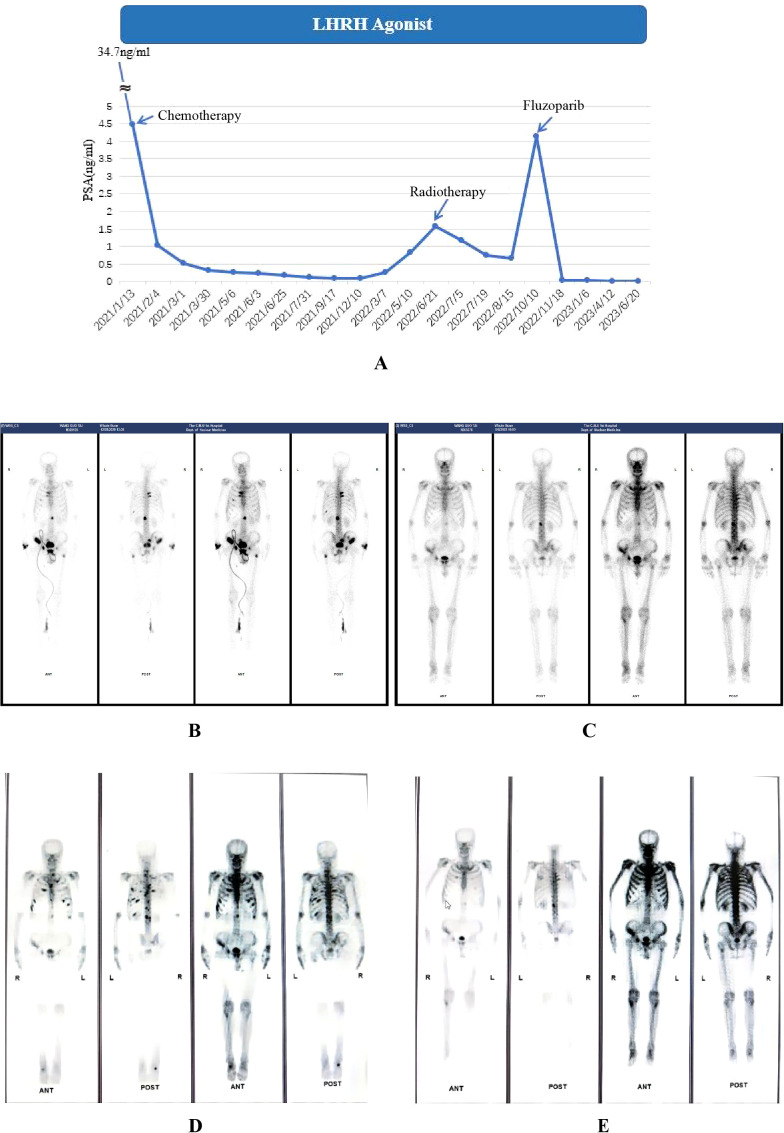
Serum PSA level **(A)** and ECT results **(B–E)** of case 2. **(A)**. LHRH: Luteinizing Hormone-Releasing Hormone. Chemotherapy: docetaxel/albumin-bound paclitaxel + cisplatin. Detailed dates and values of each PSA test can be found in the **Supplementary Table S3**; **(B)** (December 2020, at the time of diagnosis) Multiple radionuclide-concentrating foci were seen in bilateral ribs, L1 vertebrae, left femur, right iliac bone, pubic symphysis and right wrist; **(C)** (April 2022, before radiotherapy) Radionuclide-concentrating foci were detected in bilateral ribs, L1 vertebrae and right wrist. **(D)** (October 2022, before treatment of Fluzoparib) Radionuclide-concentrating foci of bilateral ribs and T2-T4、L1 vertebrae; **(E)** (April 2023, after 6-month treatment of Fluzoparib) A reduction of metastatic foci on bilateral ribs and vertebrae was seen.

In June 2021, the PSA level decreased to 0.224 ng/ml after first-line therapy, which comprised continuous ADT with goserelin (10.8 mg) and docetaxel-cisplatin (120 mg +120 mg) chemotherapy twice, followed by four doses of albumin-bound paclitaxel + cisplatin (400mg + 120mg) (**Figure 3A**). The detailed usage and dosage of the chemotherapy drugs were the same as in case 1 (**Supplementary Table S1**). From September 2021 to May 2022, PSA level continuously rose to 0.846 ng/ml. Considering the possibility of biochemical progression, radiotherapy was applied in June 2022 as second-line treatment. After detecting treatment failure in October 2022, when the PSA level climbed to 4.13 ng/ml, third-line therapy consisting of continuous ADT (goserelin,10.8mg), Fluzoparib, and abiraterone was initiated. Olaparib was recommended at the beginning, but the decision of Fluzoparib was finally made due to his poor economic status. To assess the therapeutic effect, ECT bone scans were conducted during routine follow-ups. It The PSA level decreased to 0.03 ng/mL about half a month after treatment in November 2022 and he reached PSA90. The PSA remained at low levels, and an improvement in ECT performance was also observed with a significant reduction of metastatic foci, as reported by images in April 2023 (**Figures 3B–E**).

**Figure 2B** shows the overall changes in the results of blood tests during the entire treatment process. A continuous grade 1 anemia was detected by blood tests during the whole routine follow-ups. An occasional lymphocytopenia (0.48*10^9^/L, grade 3) was found during the period of radiotherapy. Occasional thrombocytopenia occurred during chemotherapy (grade 1, once). However, during radiotherapy and PARP inhibitor treatment, grade 1 thrombocytopenia has gradually become a persistent state. It is worth noting that the patient suffered from an attack of COVID-19 in September 2023, featured by continuous high fever. At this point, the patient underwent a blood cell test and found a transient abnormal decrease in lymphocytes and platelets (grade 3) compared to before, which may be attributed to potential immune decline after receiving chemotherapy, radiation therapy, and PARP inhibitor treatment. Detailed numerical results of blood cell test were presented in **Supplementary Figures S7**-**S12**.

## 4 Discussion

In recent years, there has been a rise in the prevalence of AVPC with or without neuroendocrine differentiation characteristics among patients. This subtype of prostate cancer is characterized by tumor cells that often grow independently from the androgen receptor signal transduction pathway, losing its typical features of prostate adenocarcinoma. Histopathological and molecular features of AVPC vary on both inter- and intra-tumoral levels, indicating its heterogeneous nature. AVPC may present as small cell carcinoma displaying the typical morphology of tumor cells with scant or absent cytoplasm and nucleoli. These tumors typically express neuroendocrine (NE) markers as detected through immunohistochemistry including chromogranin A (CHGA), synaptophysin (SYP), neuron-specific enolase 2 (ENO2) and neural cell adhesion molecule 1 (NCAM1, CD56) (13). Expression of PSA and AR is frequently not detected in this type of tumor. In terms of morphology, the cell may resemble a simple small cell carcinoma, a typical prostate adenocarcinoma, or a mixed tumor containing adenocarcinoma tissue and neuroendocrine cells, which complicates diagnosis and treatment (13).

In the era of molecular medicine, genetic testing and next-generation sequencing (NGS) genomic profiling assays play a crucial role not only in the identification of AVPC from typical prostate cancer, but also in selecting potentially effective therapies, especially in cases where androgen-related therapies may fail in the treatment of AVPC. According to the guideline of National Comprehensive Cancer Network (NCCN) for prostate cancer, NGS testing has been recommended to be applied to patients with metastatic prostate cancer or prostate cancer in high-risk group in order to clarify the pathological subtypes with poor prognosis and predict the efficacy of different systemic treatments (14). A number of studies have pointed out a close relationship between specific gene mutations and different therapies. For example, the alterations of AR, TP53, RB1 and PTEN may suggest a diagnosis of AVPC and a poor response to endocrine therapies (15–17). PARP inhibitors were also reported to perform better in patients with gene alterations of BRCA 1/2 and ATM (18–21). Tumor mutation burden (TMB) measured during sequencing has also been reported to predict the therapeutic effect of PD1/PD-L1 inhibitors (22, 23). With further research on specific gene mutations, treatment plans for patients undergoing gene sequencing will become more precise and personalized in the future.

Due to the rarity, rapid progression and poor prognosis of AVPC, there is a significant challenge in treatments of AVPC, without unified recommendations from existing guidelines. Our report on these two cases with satisfactory outcomes offer valuable evidences for the real-world exploration of AVPC treatments with durable clinical benefit from PARP inhibitor + radiotherapy after failure of chemotherapy. Detailed reasons for the selection of drugs for the whole process of systemic results were presented in **Table 1**. Referring to the existing guidelines, our treatment strategies for *de novo* AVPC are as follows (**Supplementary Figure S13**). First, NGS should be recommended to patients with metastatic prostate cancer to provide information on subtype diagnosis and formation of systemic treatment protocols. Second, the continuous ADT should be applied as a background treatment during the whole process of treatment. Some parts of AVPC tissues still exhibit androgen dependence, and continuous ADT can prevent further progression to some degree (24). Novel hormone therapy (NHT) should also be considered as one of the optional therapies depending on the results of genetic testing. Third, platinum-based chemotherapy should be considered as the first-line therapy for its similar manifestations of small cell carcinoma, followed by second-line radiotherapy at the hormone-sensitive stage. Fourth, when it finally progressed into castration-resistant stage, PARP inhibitors could be a reasonable first-line therapy for patients with HRR gene mutations. However, as mentioned in NCCN guideline, further treatments are still unclear in this situation. Recommendations for patients to attend clinical trials are encouraged, and re-challenge of chemotherapy is also a proper decision. Our treatment strategy works successfully in these two reported cases after the use of PARP inhibitors and ADT, with excellent PSA control and prognosis observed for three and two years, respectively.

According to our strategy mentioned above, we choose PARP inhibitors instead of other drugs (177-Lu and PD/PD-L1 inhibitors for example) for concurrent or subsequent use with radiotherapy due to their potential synergistic effects. Several studies have reported the radiosensitizing effect of PARP inhibitor both *in vitro* and *in vivo* (25, 26). The radiosensitization is achieved possibly due to the inhibition of poly(ADP-ribose) polymerase, which plays an important role in the process of homologous recombination repair and preventing the detection of DNA single-strand breaks (SSBs). As a result, successful repair of SSBs is inhibited, leading to the accumulation of double-strand breaks (DSBs) and ultimately the death of cancer cells. At the individual level, the outcome of our two cases supported the satisfactory effect of the combination of radiotherapy and PARP inhibitors.

Our article has the following advantage. First, although *de novo* AVPC is an extremely rare subtype of prostate cancer, both of our two cases have received timely diagnosis and precise treatments, resulting in well prognosis until now. Our experience emphasized the importance of NGS testing for metastatic and high-risk cases. Second, we shared our strategies on the treatment of AVPC which showed a satisfactory control on disease progression and an acceptable safety protocol, aiming to provide useful information and experience for the management of AVPC. Third, detailed description of adverse event was presented with line charts of blood test results and treatment method timeline labels, which showed the adverse effect of different treatments in a more concise and intuitive way. However, this case report also has several limitations. The difficulties in intercity traffic and outpatient service during the epidemic of the COVID-19 prevented the patients from adhering to the doctor’s recommendations strictly for regular follow-up examinations resulting in a lack of image information. That has obstructed the radiologic assessment of disease progression and remission. Additionally, due to the poor economic conditions of case 2, he finally choose Fluzoparib as the drug for PARP inhibitor treatment, different from the original plan (Olaparib, the same with case 1), making the treatment experience less comparable between our two cases.

## 5 Conclusion

In conclusion, we present two cases of *de novo* AVPC which was diagnosed by biopsy and genetic testing and finally well-controlled after radiotherapy and PARP inhibitor treatment with sustained response in PSA and long-term survival benefits. The treatment experience of our cases reflects the importance of genetic sequencing for precise diagnoses and treatment selections. Future studies on the exploration for optimal treatment strategies for NEPC and AVPC with different gene mutations is required, especially in terms of experimental treatment options after PARP inhibitor failure.

## Data Availability

The original contributions presented in the study are included in the article/**Supplementary Material**. Further inquiries can be directed to the corresponding authors.
