# Supplementary material for: Case Report: Two cases of chemotherapy refractory aggressive variant prostate cancer with extreme durable response to PARP inhibitor
Source: Front Oncol. 2025 Apr 24;15:1533627. doi: 10.3389/fonc.2025.1533627 (PMC12058769; doi:10.3389/fonc.2025.1533627)
Supplement: Supplementary file 1 [file DataSheet1.docx]

**
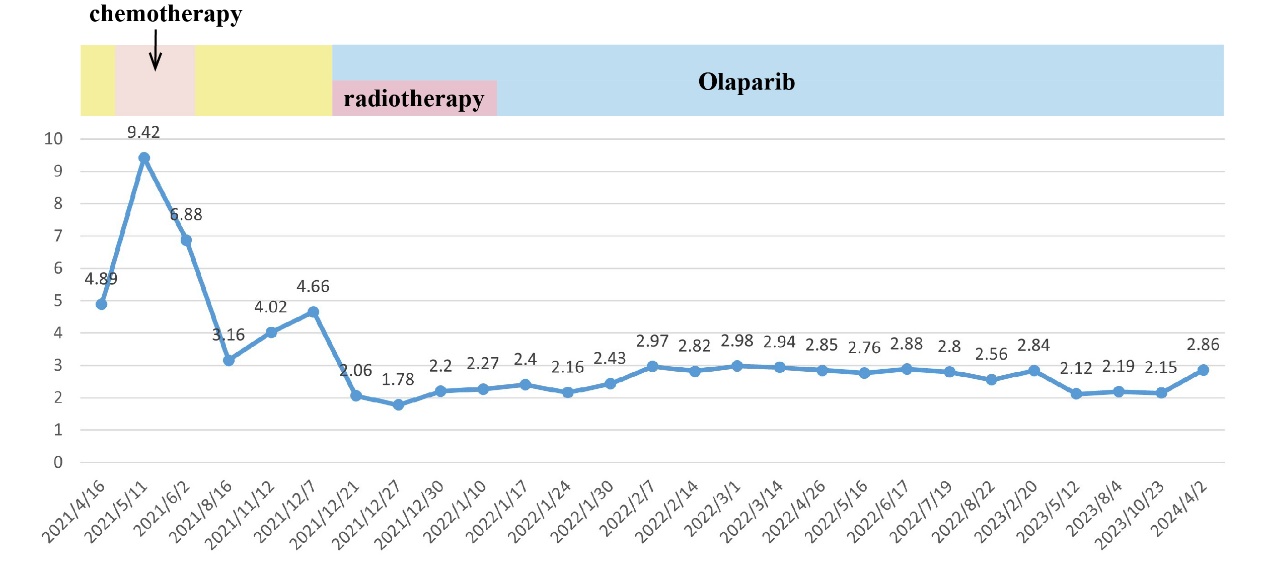
**

**Supplementary Figure S1:** Changes of granulocyte count of case 1 (unit: 10^9^/L). Normal range is 1.8×10^9^/L to 6.3×10^9^/L.


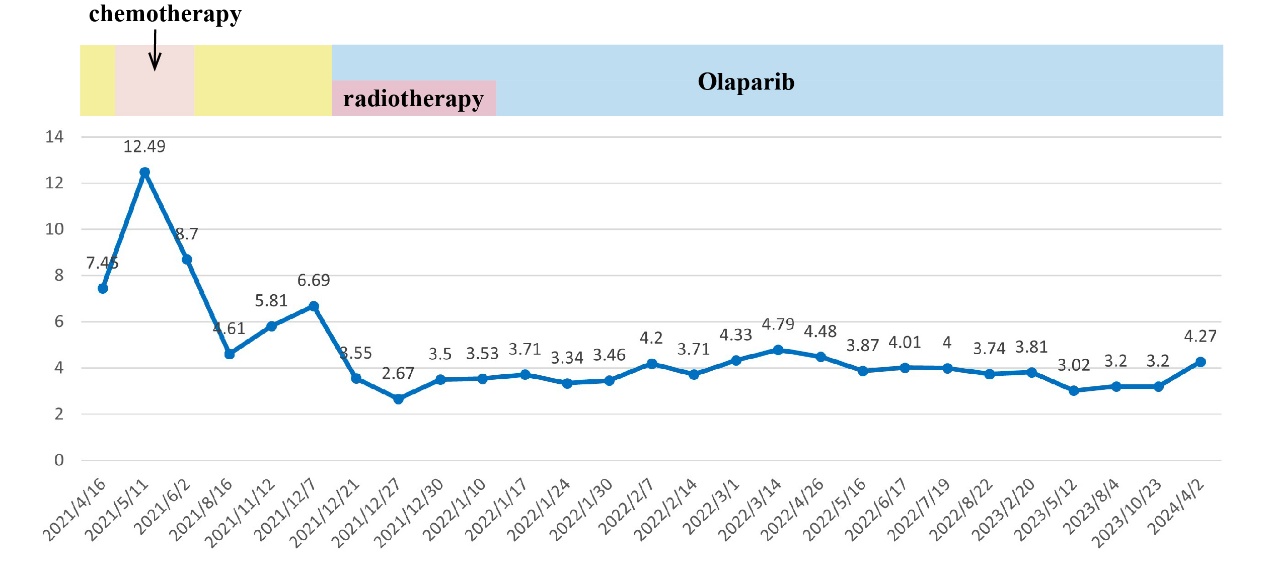


**Supplementary Figure S2:** Changes of leukocyte count of case 1 (unit: 10^9^/L). Normal range is 3.5×10^9^/L to 9.5×10^9^/L.


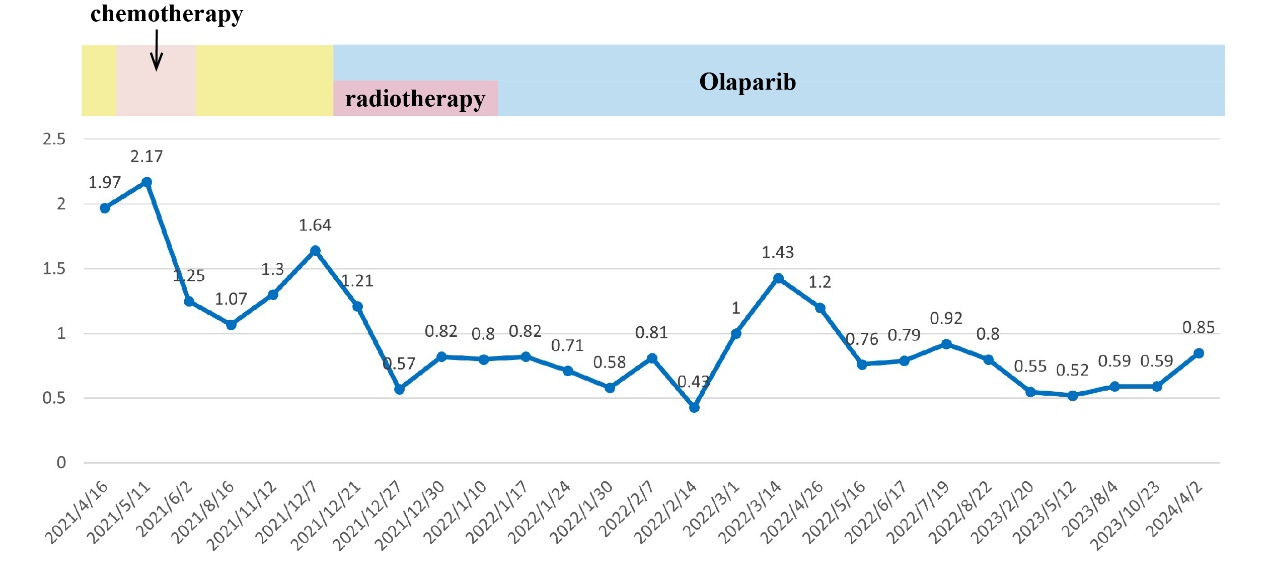


**Supplementary Figure S3:** Changes of lymphocyte count of case 1 (unit: 10^9^/L). Normal range is 1.1×10^9^/L to 3.2×10^9^/L.


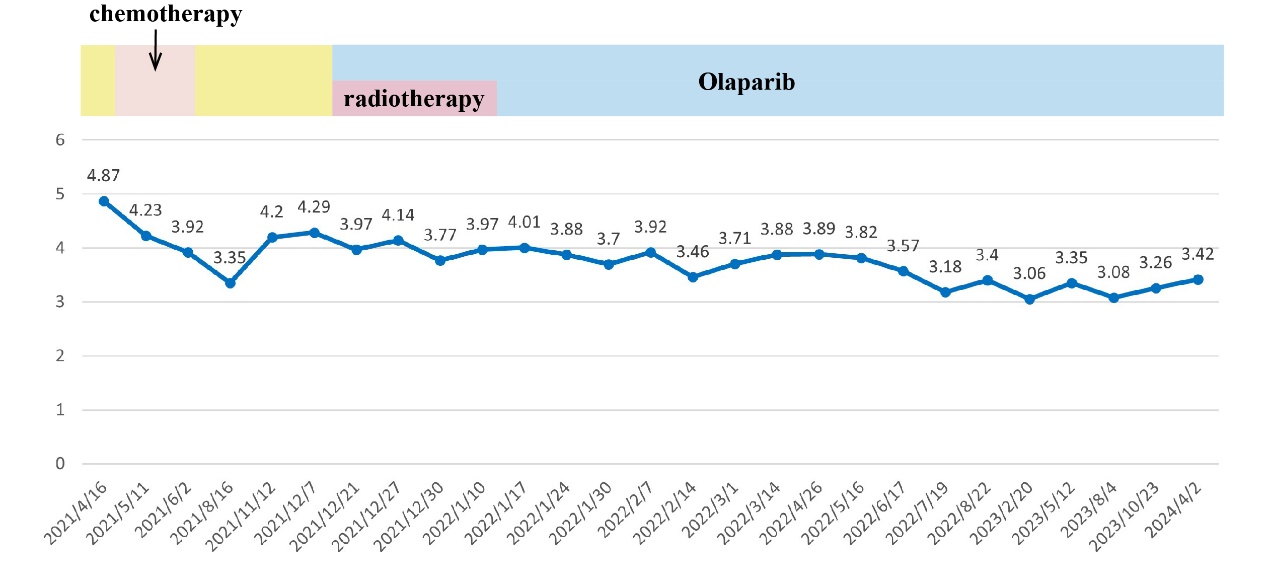


**Supplementary Figure S4:** Changes of red blood cell (RBC) count of case 1 (unit: 10^12^/L). Normal range is 3.8×10^12^/L to 5.1×10^12^/L.


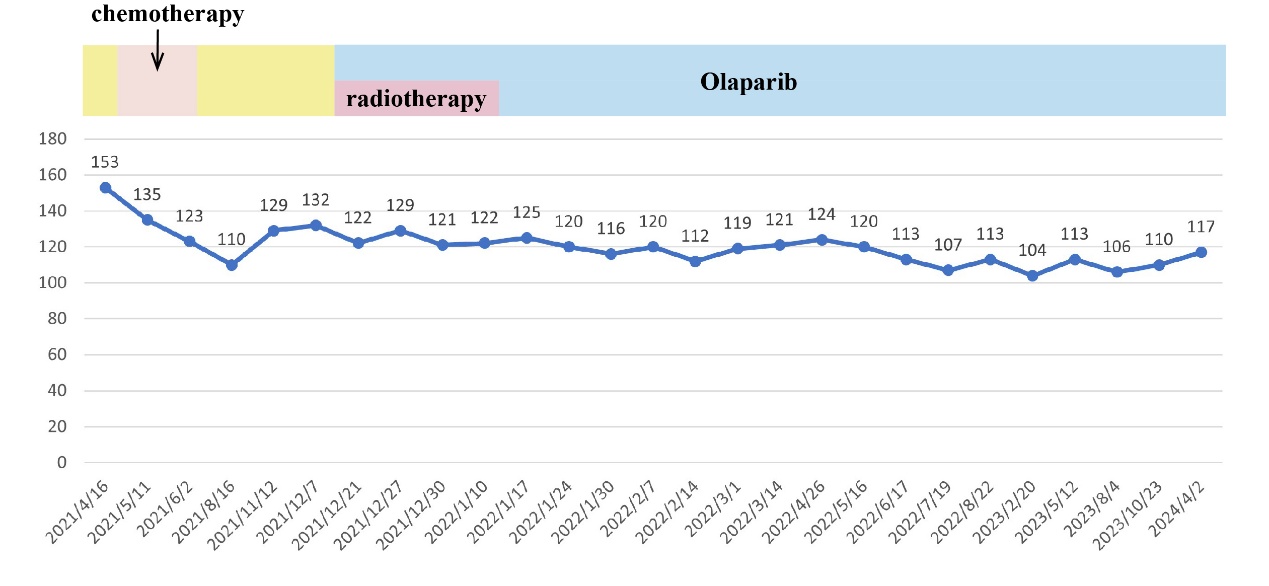


**Supplementary Figure S5:** Changes of hemoglobin level of case 1 (unit: g/L). Normal range is 115g/L to 150g/L.


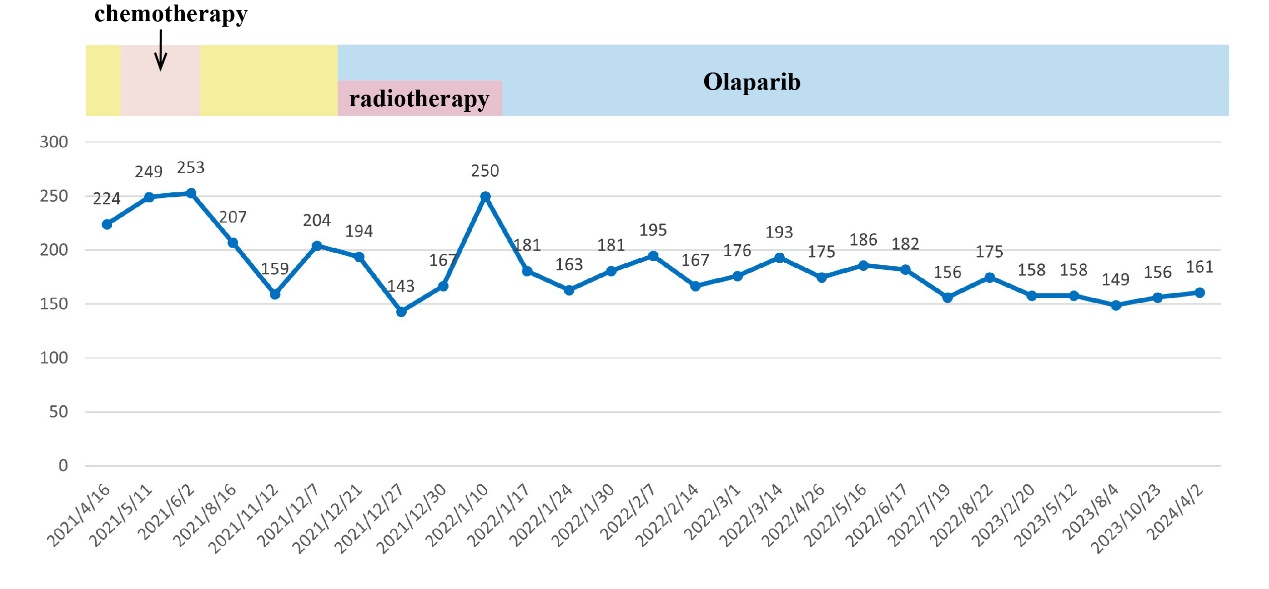


**Supplementary Figure S6:** Changes of platelet count of case 1 (unit: 10^9^/L). Normal range is 125×10^9^/L to 350×10^9^/L.


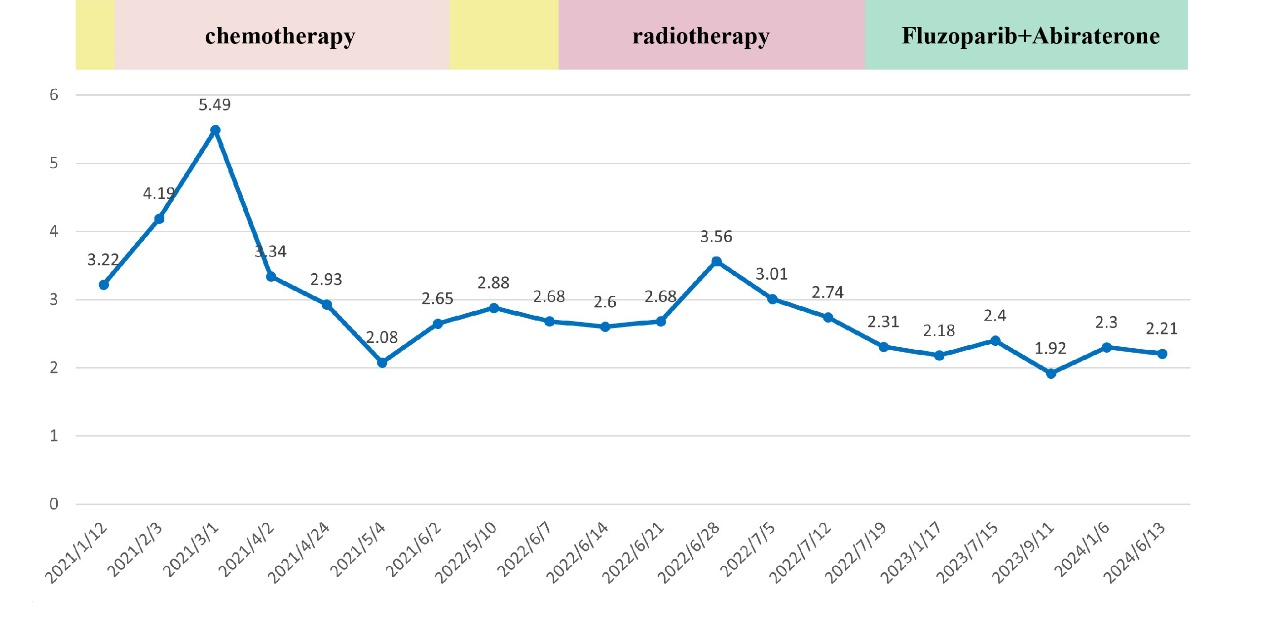


**Supplementary Figure S7:** Changes of granulocyte count of case 2 (unit: 10^9^/L). Normal range is 1.8×10^9^/L to 6.3×10^9^/L.


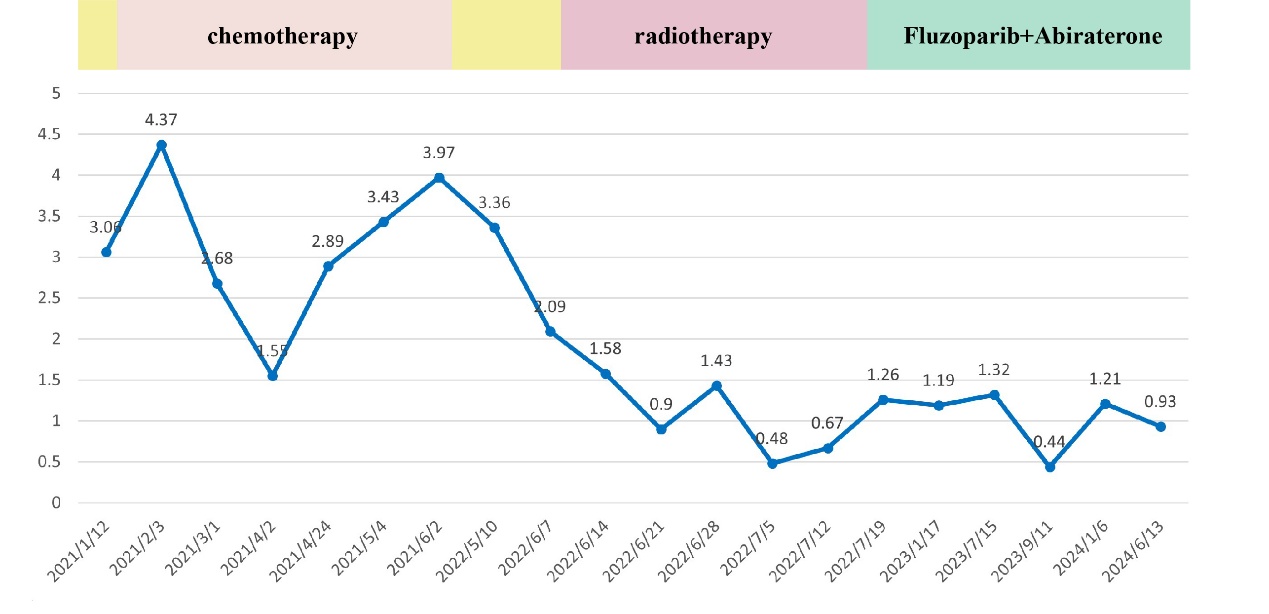


**Supplementary Figure S8:** Changes of leukocyte count of case 2 (unit: 10^9^/L). Normal range is 3.5×10^9^/L to 9.5×10^9^/L.


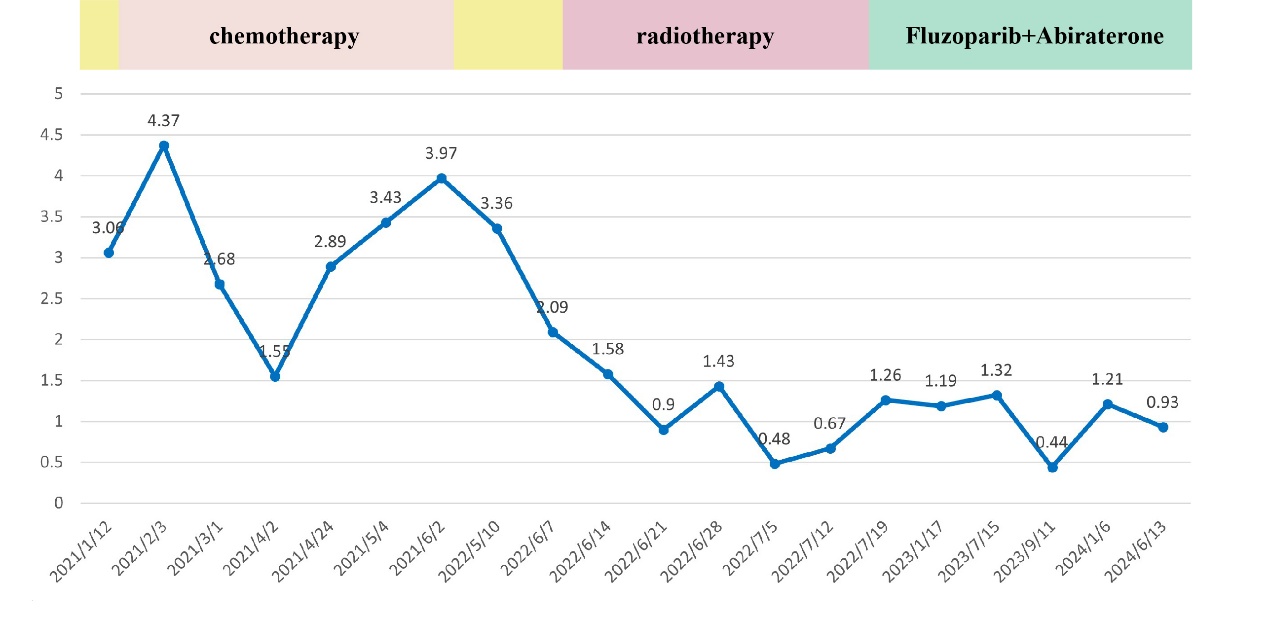


**Supplementary Figure S9:** Changes of lymphocyte count of case 2 (unit: 10^9^/L). Normal range is 1.1×10^9^/L to 3.2×10^9^/L.


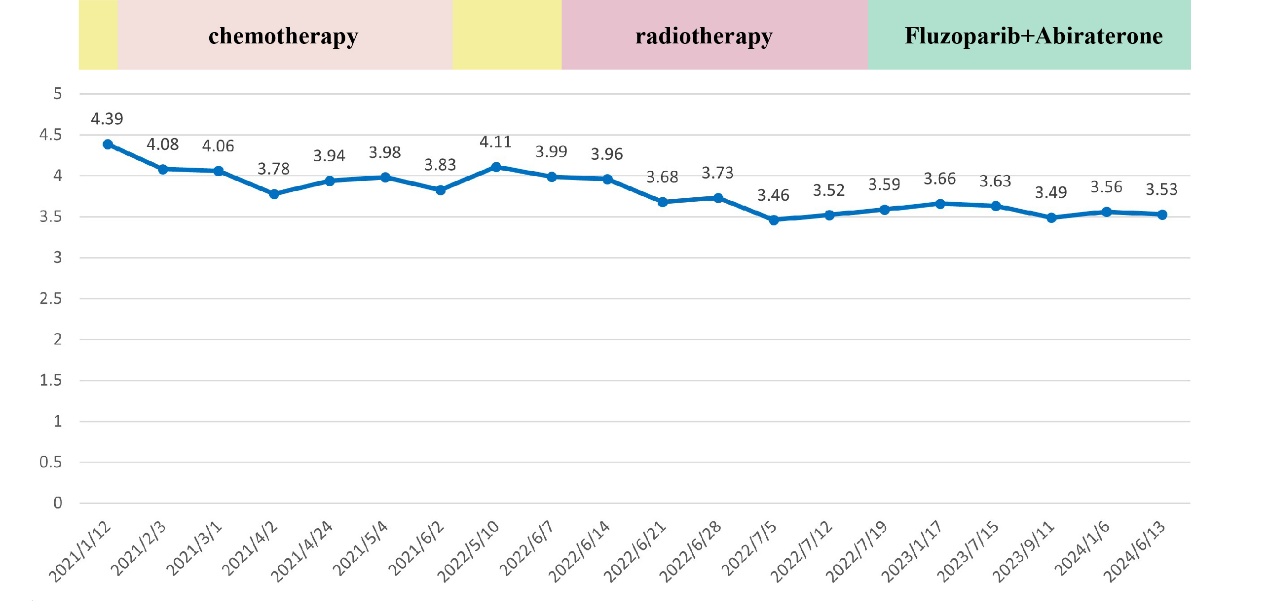


**Supplementary Figure S10:** Changes of red blood cell (RBC) count of case 2 (unit: 10^12^/L). Normal range is 3.8×10^12^/L to 5.1×10^12^/L.


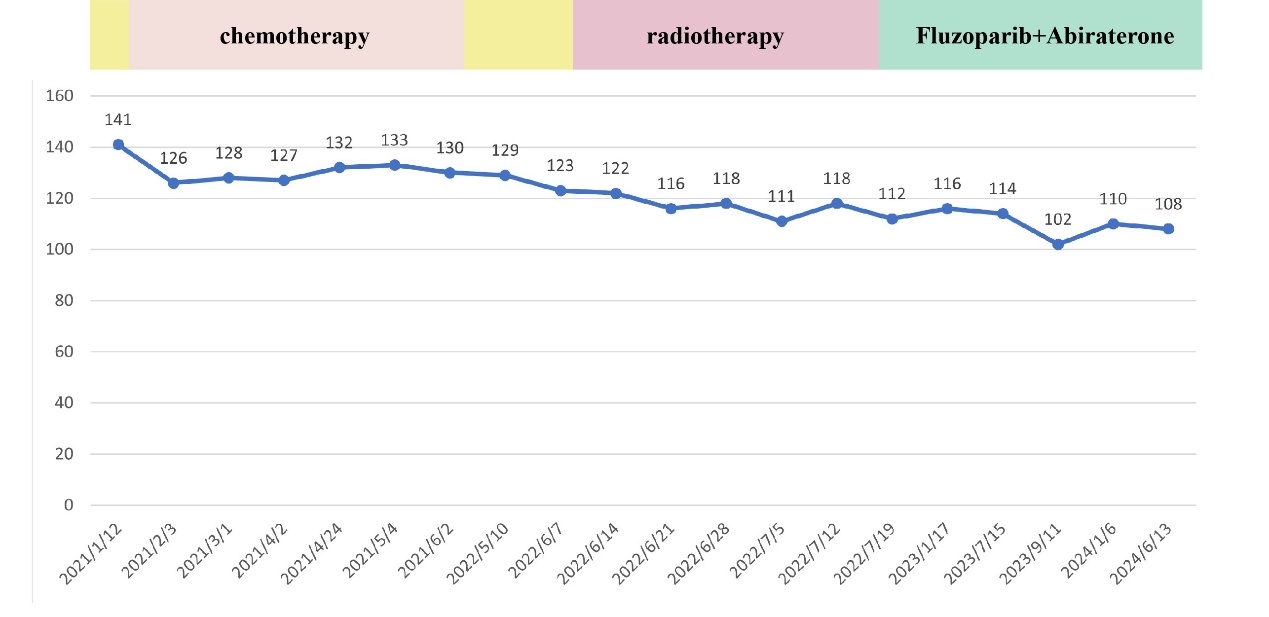


**Supplementary Figure S11:** Changes of hemoglobin level of case 2 (unit: g/L). Normal range is 115g/L to 150g/L.


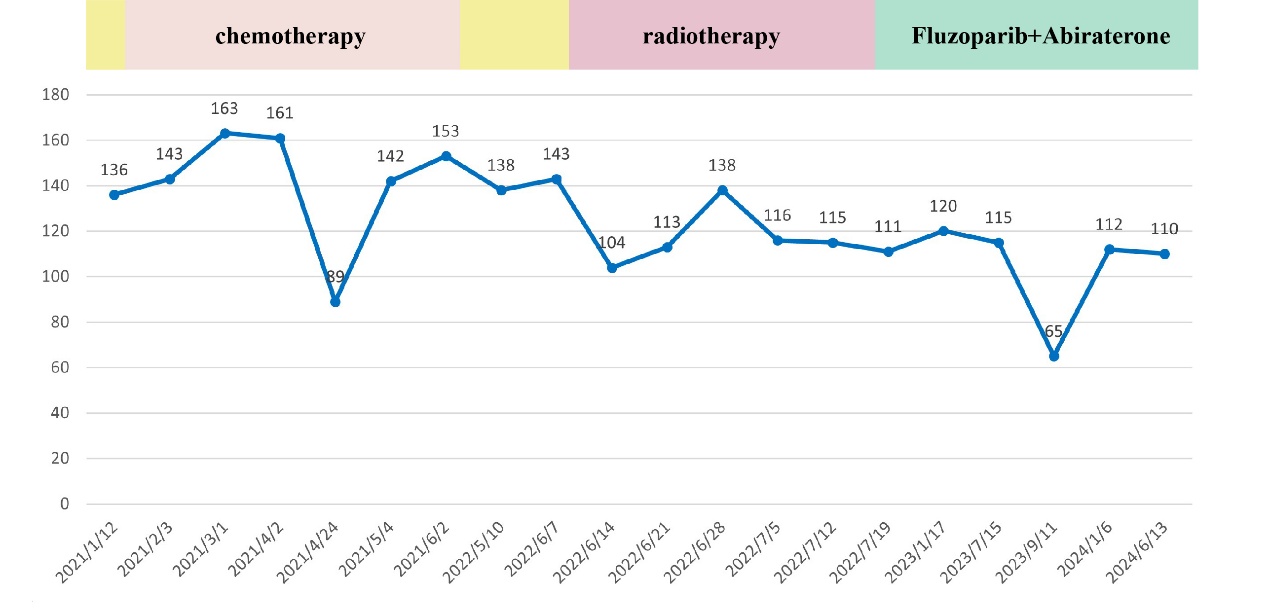


**Supplementary Figure S12:** Changes of platelet count of case 2 (unit: 10^9^/L). Normal range is 125×10^9^/L to 350×10^9^/L.


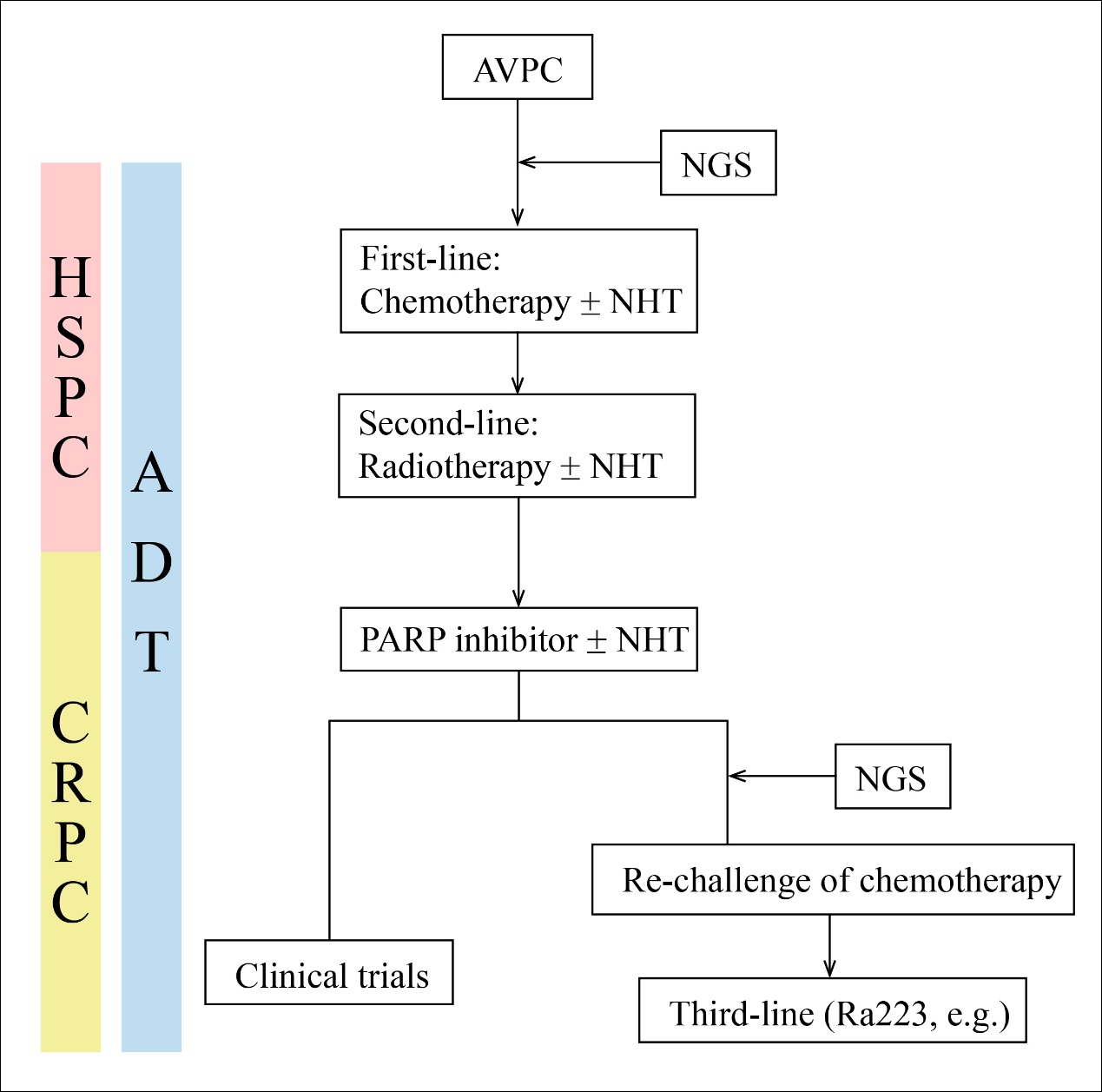


**Supplementary Figure S13. Our strategies of treatments of aggressive variant prostate cancer (AVPC).** HSPC: hormone-sensitive prostate cancer; CRPC: castration-resistant prostate cancer; ADT: Androgen Deprivation Therapy; NGS: next-generation sequencing; NHT: novel hormone therapy.

**Supplementary Table S1:** Detailed intravenous administration protocol of docetaxel/ albumin-bound paclitaxel + cisplatin.

| **Drug** | **Solvent** | **Notes** |
| --- | --- | --- |
| Docetaxel (120mg) | Normal Saline (NS) | Docetaxel is administered in two doses. First dose: docetaxel 20mg + NS 100ml; Second dose: docetaxel 100mg + NS 250ml. The two-dose administration is to reduce the possible discomfort. |
| Albumin-bound paclitaxel (400mg) | Normal Saline (NS) | Albumin-bound paclitaxel 400mg + NS 80ml. |
| Cisplatin (120mg) | Normal Saline (NS) | Cisplatin is administered in two days . First dose: Cisplatin 60mg + NS 500ml; Second dose: Cisplatin 60mg + NS 500ml. The two-dose administration is to reduce the possible discomfort. |

**Supplementary Table S2:** Detailed dates and PSA values of Case 1.

| Year/ Month/ Date | PSA Value (ng/ml) |
| --- | --- |
| 2021/4/1 | 125 |
| 2021/4/20 | 33.7 |
| 2021/5/11 | 3.88 |
| 2021/6/1 | 0.946 |
| 2021/6/24 | 0.485 |
| 2021/7/16 | 0.265 |
| 2021/8/16 | 0.151 |
| 2021/9/15 | 0.15 |
| 2021/10/11 | 0.299 |
| 2021/11/12 | 0.496 |
| 2021/12/7 | 0.647 |
| 2021/12/27 | 0.469 |
| 2022/1/10 | 0.237 |
| 2022/1/24 | 0.212 |
| 2022/2/7 | 0.142 |
| 2022/2/14 | 0.13 |
| 2022/3/1 | 0.113 |
| 2022/3/14 | 0.096 |
| 2022/4/26 | 0.071 |
| 2022/5/16 | 0.067 |
| 2022/5/23 | 0.063 |
| 2022/6/17 | 0.046 |
| 2022/7/19 | 0.036 |
| 2022/8/22 | 0.033 |
| 2022/10/12 | 0.041 |
| 2022/11/21 | 0.04 |
| 2023/2/20 | 0.019 |
| 2023/5/12 | 0.022 |
| 2023/8/4 | 0.016 |
| 2024/1/10 | 0.018 |
| 2024/4/2 | 0.02 |
| 2024/6/20 | 0.015 |

**Supplementary Table S3:** Detailed dates and PSA values of Case 2.

| Year/ Month/ Date | PSA Value (ng/ml) |
| --- | --- |
| 2020/12/14 | 34.7 |
| 2021/1/13 | 4.49 |
| 2021/2/4 | 1.04 |
| 2021/3/1 | 0.51 |
| 2021/3/30 | 0.316 |
| 2021/5/6 | 0.252 |
| 2021/6/3 | 0.224 |
| 2021/6/25 | 0.179 |
| 2021/7/31 | 0.116 |
| 2021/9/17 | 0.094 |
| 2021/12/10 | 0.104 |
| 2022/3/7 | 0.26 |
| 2022/5/10 | 0.846 |
| 2022/6/21 | 1.57 |
| 2022/7/5 | 1.18 |
| 2022/7/19 | 0.74 |
| 2022/8/15 | 0.65 |
| 2022/10/10 | 4.13 |
| 2022/11/18 | 0.03 |
| 2023/1/6 | 0.04 |
| 2023/4/12 | 0.02 |
